# Recent Advances in Understanding the Role of Genomic and Epigenomic Factors in Noncommunicable Diseases

**DOI:** 10.1155/2019/1649873

**Published:** 2019-04-08

**Authors:** Hossain U. Shekhar, Sajib Chakraborty, Kaiissar Mannoor, Altaf H. Sarker

**Affiliations:** ^1^Department of Biochemistry and Molecular Biology, University of Dhaka, Bangladesh; ^2^Genetics and Genomics Laboratory Institute for Developing Science and Health Initiatives, Bangladesh; ^3^Lawrence Berkeley Lab, USA

The NGS based whole genome and exome sequencing endeavors have identified a plethora of mutations in the coding regions of the human genome that are held responsible as the causative factors for various human noncommunicable diseases including cancer. These mutations encompass various nonsynonymous nucleotide substitutions causing missense, nonsense, and frameshift changes in the protein-coding genes. For these types of mutations it is easier to establish a causal link between these mutations and disease phenotypes as they tend to change the amino acids in the proteins resulting in their loss-of-function (of tumor suppressor genes) or gain-of-function (of oncogenes) which might be directly responsible for the disease such as cancer. Apart from these disease-causing mutations, vast majority (more than 80%) of single-nucleotide polymorphisms were distributed throughout the human genome, mostly in the noncoding regions such as introns and intergenic regions [1]. Large body of evidence suggested that variants in the noncoding regions can be considered as genetic predisposition to many noncommunicable diseases including cancer [2]. For instance, genome-wide association studies (GWAS) have provided statistical evidence that numerous single-nucleotide variants/polymorphisms (SNVs/SNPs) in the noncoding regions are associated with increased risk of complex diseases [3]. The direct causal relationship between allelic variants originating from SNPs and disease phenotype becomes obscure when the variation occurs in noncoding regions. Initially it was suggested that the risk variants identified by GWAS may possibly be physically associated with a neighboring protein-coding gene that is the true causative variant. However another plausible explanation is that genetic variations residing in the functional but noncoding regions may have an impact on nearby genes. Recent GWAS studies have revealed that the SNPs that are associated with diseases are preferentially concentrated in the noncoding but functional genomic regions such as enhancer elements, DNase hypersensitivity regions, and epigenetically important chromatin marks [4]. A recent study highlighted the importance of the SNPs in the noncoding regions of human genome and their functional consequences in terms of genetic propensity to cancer. This seminal study conducted by Hua* et al.* sought to uncover the underlying mechanism of prostate cancer risk-associated SNP (rs11672691) in the promoter region of a long noncoding RNA (LncRNA) [5]. SNP-rs11672691 resides in the promoter region of the short isoform of the lncRNA-PCAT19 (PACT19-short). This short form of the lncRNA is encoded by the third intron of the long isoform of lncRNA-PCAT19 (PACT19-long) gene. The allelic risk-variant due to SNP-rs11672691 is typically associated with reduced and elevated expression of short and long isoforms, respectively. The region harboring the risk-SNP has both promoter and enhancers capability and thereby considered as bifunctional. The SNP-rs11672691 in this region hinders the physical interaction of the transcription factor NKX3.1 and YY1 to the promoter of PCAT19-short leading to the diminished promoter activity without losing its functional capacity as enhancer for PCAT19-long. As a consequence the decreased and increased expression of PCAT19-short and PCAT19-long, respectively, occur in the individuals who are at risk of prostate cancer since PCAT19-long in concert with other genes transcriptionally induces certain cell cycle genes which in turn increase the risk of prostate cancer [5]. This study not only highlights the importance of the SNPs in the noncoding regions but also mechanistically shows how a single SNP can control the expression levels of two functional isoforms of lncRNAs.

This special issue underscored the roles of many genetic factors in the predisposition of variety of diseases including cancer, stroke, and cerebrovascular diseases and highlighted the influence of epigenetic modifications in kidney transplantation. For instance,* C.-C. Wu et al.* determined the genetic predisposition of urothelial carcinoma due to SNPs of inflammation associated genes, TNF-*α* and IL-8. Following the similar argument, in a review paper* J. Lin and W. Sheng *summarized the association of NF213 variant diversity with cerebrovascular diseases. Following the same line, Mst. N. Begum et al. revealed how the substitutions mutations (SNPs) in thyroid peroxidase (TPO) gene contribute to the thyroid dyshormonogenesis (TDH) in Bangladeshi patients. Apart from the uncovering the variations in genetic level,* A. Agodi *et al. emphasized the clinical potential of epigenetic modification in the form of DNA methylation in chronic kidney disease (CKD) and complications after kidney transplantation. In this review article the authors hypothesized that identifying DNA-methylation status in patients undergoing kidney transplantation has the potential to develop key strategies to prevent as well as treat the complications that typically arise from kidney transplantation procedure. In this special issue two of the published studies conducted by* S. K. Sarker* et al. and* X. Gou* et al. employed metabolomics methods to identify markers for inborn errors of metabolisms (IEMs) and drug-mechanism, respectively. Apart from these studies* M. F. Reza* et al. identified that a QTL region residing on chromosome 18 may play a role in modulating the response towards salt-induced renal injury in a blood pressure independent mechanism.* S. Dima* et al. conducted an epidemiological study to those lung cancer patients with preexisting comorbidity although it may result in the early diagnosis of lung cancer; adverse consequences of comorbidity may present a challenge in the treatment of older cancer patients. M. I. M.* Choudhury* et al. performed a similar epidemiological study to identify the incidence rate of cutaneous malignancy in Bangladesh. In a comprehensive review article,* A. Maugeri* et al. presented a detailed summary regarding the role of complement system and its genetic variants in “age-related macular degeneration (AMD)”, emphasizing the modulatory role of the interplays between genetic and environmental factors and their consequences on AMD onset, progression, and therapeutic response. In a comprehensive review,* S. Chakraborty* et al. argued in favor of multi-OMICS approaches instead of single OMICS study for cancer research. In the article the authors highlighted several examples where multi-OMICS studies were used to dissect the cellular response to chemo- or immunotherapy as well as discover molecular candidates for cancer. In summary the authors focused on the application of different multi-OMICS approaches in the field of cancer research and discussed how these approaches are shaping the field of personalized oncomedicine.

In light of the published articles in this special issue, it can be safely assumed that genetic variants as well as epigenetic factors should be investigated in-depth to understand their biological functions in human noncommunicable diseases. Study of the factors in both genomic and epigenomic levels may facilitate the discovery of biomarkers to assess the risk, diagnosis, and prognosis of noncommunicable diseases.
